# Resolutions on the Deaths of Drs. Wm. H. Atkinson and John Stephan

**Published:** 1891-10

**Authors:** 


### Resolutions Passed by the Northern Ohio Dental Association on the Deaths of

DR. WM. H. ATKINSON,
DR. JOHN STEPHAN:

By the irrevocable decree of the Ruler of the Universe, the Northern Ohio Dental Association mourns the loss of one of its charter and honorary members, Wm. H. Atkinson, M.D., D.D. S., having departed this life on April 2, 1891, in New York city.

Also John Stephan, D D.S , an active member, who died June 25, 1890, at Cleveland, O.

In the decease of the former this Society and the profession at large have lost one who was ever most keenly alive to the interests of the profession and a pioneer and leader in the science and art of dentistry.

In the death of the latter this Society loses one of its most active and conscientious members.

Committee: C. R. Butler, J. F. Siddall, T. S. Whitslar, Chas. Buffett, Henry Barnes, H. F. Harvey.

### In Memoriam.

Dr. W. H. Atkinson.—In the providence of an All-Wise and Over-Ruling Father the subject of this tribute was removed from this to a higher life at his home in New York, April 2nd, 1891.

It is eminently proper that there should be placed upon the records of this association some evidence of the esteem and appreciation entertained by the members of this body, for Dr. Atkinson.

He was one of the founders of this association, and none labored with a more persevering industry, than he, for its permanent establishment and enduring welfare. He was always present at its meetings, and ever ready with a hearty willingness to fulfil any duty, or perform any work assigned to him; he doubtless exercised a deeper and broader impress upon it than any other member; he was always the advocate of a broad and liberal policy, impatient with the low, narrow and selfish. He possessed strong convictions, and was ever ready to avow and defend them, mere antagonism added strength to his forceful nature, notwithstanding this, he would yield with child-like simplicity to evidence and reason, when properly presented.

He was not only interested here, but in association work everywhere and gave aid and co-operation in the organization and maintenance of dental societies throughout this country. He was enthusiastic in the formation of dental and scientific societies, throwing his whole energy, whenever opportunity offered, into such enterprises.

He was also greatly interested in the subject of dental education, ever ready to give wise counsel and aid, whenever it was in his power, and wherever needed.

By his enthusiasm he awakened interest, and stimulated thought wherever he went, indeed, his presence was an inspiration. He exercised an almost unparalleled influence in the profession, and that too in the way of aiding, and making better professionally those who came within its sphere, this he did at home, in his office, and abroad. He was the first to promulgate many new points in practice; he never hesitated to put forth any thing that he thought would be of service to others; he communicated freely all he had. He possessed a wonderful faculty for communicating knowledge to others, as was shown in the fact that for years he had private classes that came to his office at stated times, and sat under his instruction, and such was his power in this work, that he was able to communicate not only of his knowledge, but of his enthusiasm as well, to those who were his pupils. Every thing he did and every resource he possessed were made subservient to his ambition for the advancement of dental science and art..

He had a broad, generous and sympathetic nature; with a heart large enough for the reception of all who had any just claim upon the regard and esteem of our common humanity. He was a firm and abiding friend, sympathetic, kind, and always ready to aid those in trouble.

And now in view of this great loss,

*Resolved*, That we will ever cherish, and will seek to perpetuate the memory of our departed brother, whose demise we so sadly mourn to-day; that we will not only cherish it oursevles, but will seek to bear it on to those who come after.

*Resolved*, That in all the traits of this grand character, as above delineated, there is an example to which we can with profit conform; and especially may the younger, and the coming members of the profession be directed to this great exemplar.

*Resolved*, That this tribute of regard and affection be spread on a memorial page of the transactions of this body.

*Resolved,* That a copy, properly prepared, be sent to the family of the deceased.

*Resolved,* That a copy be sent to the dental journals of this and other countries for publication.

J. Taft,
Geo. W. McElhaney.
L. Jack,
F. Abbott,
Eugene S. Talbot.

